# Successful treatment of acquired von Willebrand syndrome associated with monoclonal gammopathy: Breaking a dangerous bond

**DOI:** 10.1007/s00508-022-02012-3

**Published:** 2022-03-19

**Authors:** Georg Jeryczynski, Hermine Agis, Sabine Eichinger-Hasenauer, Maria Theresa Krauth

**Affiliations:** 1 Division of Oncology, Department of Medicine I, Medical University of Vienna, Vienna, Austria; 2 Division of Hematology and Hemostaseology, Department of Medicine I, Medical University of Vienna, Waehringer Guertel 18–20, 1090 Vienna, Austria

**Keywords:** Acquired von-Willebrand-syndrome, Monoclonal gammopathy of undetermined significance, Antimyeloma treatment, MGUS, Autologous stem cell transplant

## Abstract

Acquired von Willebrand syndrome is exceedingly rare and accounts for only 1–3% of von Willebrand disease cases. In this short report, we present our own cases of acquired von Willebrand syndrome associated with monoclonal gammopathy. Both cases went into complete and sustained remission after intensive antimyeloma treatment. The first patient was not deemed fit for autologous stem cell transplantation and was managed with an extensive multidrug combination including daratumumab, carfilzomib, lenalidomide, cyclophosphamide and dexamethasone. After at least VGPR was achieved the coagulation studies rapidly normalized and remained normal after treatment de-escalation to lenalidomide/dexamethasone maintenance. The second patient successfully underwent ASCT after 5 cycles of induction with daratumumab, bortezomib, cyclophosphamide and dexamethasone and has remained in full hematologic and hemostaseologic remission ever since.

The two cases highlight the efficacy of aggressive antimyeloma treatment in monoclonal gammopathy-associated acquired von Willebrand syndrome to achieve normalization of coagulation study, providing a possible way to manage these patients.

## Introduction

Acquired von Willebrand syndrome (avWS) is exceedingly rare and accounts for only 1–3% of von Willebrand disease cases [1]. Among those, avWS secondary to lymphoproliferative neoplasms including monoclonal gammopathy of undetermined significance (MGUS), smoldering multiple myeloma (SMM), multiple myeloma (MM), or Waldenströmʼs macroglobulinemia is the largest group [2].

The underlying pathogenetic mechanisms of monoclonal gammopathy-associated avWS (MG-avWS) are complex and incompletely understood. They include rapid sequestration especially of large and ultra-large von Willebrand factor (vWF) multimers after binding to monoclonal antibodies [2, 3]. Detection of these antibodies is sometimes possible [4]; however, specific tests are not available.

In the setting of avWS due to MGUS, which represents the majority of patients with MG-avWS, treatment has for many years focused on approaches specific for the bleeding diathesis, including desmopressin analogues (DDVAP), vWF concentrates and high-dose intravenous immunoglobulin (IVIG) [5–7]. While the coagulation parameters after administration of IVIG may improve for up to 3 weeks, vWF concentrates and DDVAP only produce short-lived responses up to 24 h. Several case series have demonstrated their effectiveness to prevent major bleeding complications during invasive procedures [3, 5, 8–10]. It is known, however, that avWS in patients with lymphoproliferative neoplasms can be improved by treatment directed against the malignant clone. While in patients with end-organ damage related to MM, immediate treatment is required and recommended according to international guidelines, the approach in avWS patients with MGUS or SMM without end-organ damage or formal treatment indications is less clear [11]. Several groups reported improvement or even normalization of MG-avWS following antimyeloma treatment [12–14].

Here, we present two patients with MG-avWS who were successfully treated with antimyeloma treatment. While both patients had a history of spontaneous and trauma-associated bleeding events, the extent and severity of the bleeding disorder differed. They were counseled on their condition and the possible association with the underlying MG. Despite not fulfilling the international myeloma working group criteria for MM, both opted for the initiation of antimyeloma treatment. They provided informed consent and were aware of the experimental nature of this approach. Baseline characteristics at the time of diagnosis of MG-avWS, interventions and outcomes are provided in Table 1.

**Table 1:** Baseline patient characteristics and interventions

	Patient No. 1 71 years, male	Patient No. 2 59 years, male	Normal range
Paraprotein Type of monoclonal gammopathy	IgG-kappa SMM	IgG-kappa Solitary plasmacytoma with minimal marrow involvement	–
Bone marrow plasma cells (in %)	50	5	–
aPTT	50.9 s	42.6 s	27.0–41.0 s
FVIII activity (in %)	14	15	60–230
vWF:Ag activity (in %)	10	17	60–180
vWF:Act (in %)	4	21	48–170
vWF:Rco (in %)	10	< 10	60–180
vWF multimer studies	No large multimers present	Normal distribution pattern	–
Bleeding symptoms	Extensive bleeding history including life-threatening hemorrhagic shock during mechanical ventilation	Perisurgical hematoma following surgery for pathological femoral fracture	–
Intervention	Antimyeloma treatment	Antimyeloma treatment and ASCT	–
Outcome	Complete normalization of coagulation studies, no further bleeding complications	Complete normalization of coagulation studies, no further bleeding complications	–

*MGUS* monoclonal gammopathy of undetermined significance, *SMM* smoldering multiple myeloma, *aPTT* activated partial thromboplastin time, *FVIII* factor VIII, *vWF:Ag* von Willebrand factor antigen, *vWF:Act* von Willebrand factor activity, *vWF:RCo* von Willebrand factor ristocetin cofactor activity

## Patient 1

**Fig. 1:**
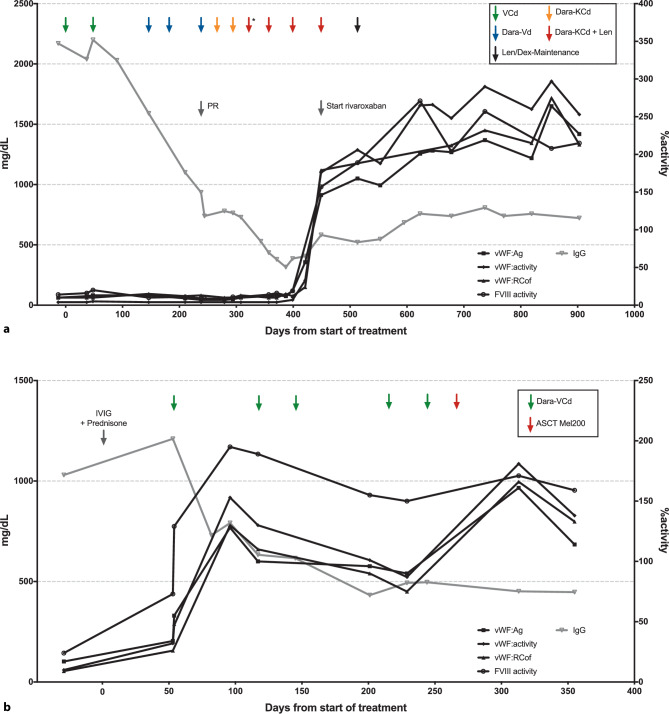
Treatment course of patients 1 (**a**) and 2 (**b**). The first cycle was administered without cyclophosphamide. *VCd* bortezomib, cyclophosphamide, dexamethasone, *Dara-Vd* daratumumab, bortezomib, dexamethasone, *Dara-KCd* daratumumab, carfilzomib, cyclophosphamide, dexathemasone, *Len* lenalidomide, *Dara-VCd* daratumumab, bortezomib, cyclophosphamide, dexamethasone, *Mel200* melphalan 200 mg/m^2^

Patient 1 is a 71-year-old man with recurring episodes of massive epistaxis as well as bleeding episodes following surgical procedures since 1995. Initially, no abnormalities in coagulation studies could be identified. In 2001, at the age of 54 years the patient was referred to our department after a severe bleeding episode following elective arthroscopy. At that time vWF antigen and factor VIII (FVIII) activity were 20% and 32%, respectively. Without evidence of bleeding before 1995, avWS was suspected and IgG-kappa paraproteinemia was detected. In the absence of any end-organ damage and because of a low and stable M‑gradient, bone marrow (BM) biopsy was initially deferred until 2005 when IgG levels increased (5–10% monoclonal BM plasma cells). Meanwhile, the patient was managed with recombinant FVIII/vWF before interventions, spontaneous epistaxis episodes, however, continued. In 2011 an almost fatal pulmonary hemorrhage occurred during severe pneumonia. In 2018, when BM biopsy was repeated because of progressive paraproteinemia, 50% clonal plasma cells were detected. The IgG levels were below 3 g/dl, and the free light chain ratio was 4. In the absence of any end-organ damage, a diagnosis of SMM was established. Given the continued bleeding complications, including a one-time almost fatal event, the patient opted for initiation of antimyeloma treatment to eradicate the potentially causative paraprotein. He received two cycles of a standard induction regimen with bortezomib/cyclophosphamide/dexamethasone (VCD; bortezomib 1.3 mg/m^2^ s.c., cyclophosphamide 300 mg/m^2^ i.v., dexamethasone 20 mg p.o. on days 1, 8, 15 and 22) but paraprotein levels and coagulation parameters remained unchanged (Fig. 1a). Treatment was switched to daratumumab/bortezomib/dexamethasone (daratumumab 16 mg/kg i.v. on days 1, 8, 15, 22 for the first 2 cycles, and days 1 and 15 thereafter), which after 4 cycles resulted in a partial response but without effect on coagulation parameters. Treatment was intensified by reintroducing cyclophosphamide and replacing bortezomib with carfilzomib (20 mg/m^2^ i.v. on days 1, 8 and 15). While the hematological response slowly deepened, the coagulation abnormalities persisted. Therefore, lenalidomide was introduced at 15 mg (days 1–21) in addition to daratumumab/carfilzomib/cyclophosphamide/dexamethasone. With this regimen, serum electrophoresis normalized, serum and urine immunofixation became negative and vWF-specific coagulation markers normalized within less than 3 weeks. Lenalidomide was later increased to 20 mg (days 1–21). After sustained response (VGPR or even CR), the therapy was de-escalated to lenalidomide/dexamethasone maintenance. The BM biopsy for response evaluation was postponed because of severe low back pain with indications for spinal surgery. At the last follow-up, 10 months after therapy de-escalation, the patient remained in CR/VGPR with negative serum and urine immunofixation and normal coagulation parameters. He successfully underwent spinal surgery without any bleeding complications or need for substitution of vWF/FVIII concentrates.

## Patient 2

Patient 2 is a 59-year-old male patient who was diagnosed with solitary plasmocytoma in the left femur that led to a pathological fracture in November 2019. During the surgical resection of the osteolytic lesion, he developed life-threatening bleeding. Medical history and coagulation parameters were consistent with avWS. Minimal serum paraprotein was detectable (only serum immunofixation positive). Further BM examination showed minimal involvement (5% clonal plasma cells) without indications for systemic treatment. In May 2020, a spontaneous, severe hemorrhoidal hemorrhage occurred, which was successfully treated with IVIG and prednisolone. After counseling about the underlying MG-avWS, the patient opted for an induction therapy with subsequent autologous stem cell transplantation (ASCT). To avoid prophylactic anticoagulation, daratumumab/bortezomib/cyclophosphamide and dexamethasone as an immunomodulatory-free regimen was chosen (initially daratumumab at 16 mg/kg i.v. on days 1, 8, 15, 22 for 1 cycle, then at 1800 mg s.c. for the following cycles, from cycle 3 onwards daratumumab was only given on days 1 and 15; bortezomib 1.3 mg/m^2^ s.c., cyclophosphamide 300 mg/m^2^ i.v., dexamethasone 20 mg p.o. on days 1, 8, 15 and 22). After the first cycle coagulation parameters normalized (Fig. 1b) in parallel to serum immunofixation negativity. After completion of 6 cycles, ASCT with melphalan 200 mg/m^2^ was performed without complications, and the patient reached a complete response.

## Discussion

In summary, both patients treated with clonal directed therapy showed a normalization of coagulation tests through eradication of the paraprotein. These findings serve as indirect proof of a causal relationship between the clonal paraprotein and the clinically significant bleeding disorder characterized as avWS.

To date, no standard treatment guidelines are available for this distinct and rare disorder. As for inherited vWS, DDAVP and factor replacement is recommended but results in only very short-lasting effects. The IVIG is also effective with a slightly longer response duration. None of these interventions, however, can induce a stable and durable response because they have no effect on the pathomechanisms of the bleeding disorder.

In the case of avWS-MGUS, a potentially reversible and even curable underlying condition is present, since the monoclonal paraprotein is the primary cause of the disease. Therefore, targeting the malignant plasma-cell clone as the root of evil seems to be of major importance; however, as reflected by both cases partial elimination of the disease-initiating clone seems insufficient. The persistence of paraprotein is associated with persistence of avWS and even small amounts of the paraprotein maintain the pathologic process. Only the full eradication of the clone with undetectable paraprotein levels led to durable responses with complete resolution of the bleeding disorder.

A number of recently published cases reported encouraging results with clonal directed treatment [12–14]; however, the experience from these cases showed that remissions were less likely to be durable if single drug regimens were used or if the treatment was discontinued prematurely, i.e. before the causative plasma cell clone was sufficiently eradicated.

Based on these observations, we favor an approach similar to overt MM, where induction therapy is central and should be an intensive multidrug combination-treatment. In recent years, even quadruplet regimens have been tested in phase 3 trials. The aim of defining the most effective first-line therapy is the induction of a long-lasting remission. This holds true in MM and likely in MG with clinical significance. In selected cases, the eradication of a plasma cell clone although rather small could possibly represent a curative approach.

In summary, we successfully treated two avWS-MG patients with intensive but risk-adapted multidrug regimens and eradicated the malignant plasma cell clone. Consequently, the life-threatening MG-related bleeding disorder completely resolved in both patients. Based on our findings, complete eradication of the malignant plasma cell clone seems to be of pivotal importance to achieve durable responses or possibly even cure of the potentially life-threatening bleeding disorder. Given the rarity of the disease we encourage international collaborations for generating larger data sets and prospective clinical trials to build upon our findings.
